# Implementing an intervention to improve decision making around referral and admission to intensive care: Results of feasibility testing in three NHS hospitals

**DOI:** 10.1111/jep.13167

**Published:** 2019-05-17

**Authors:** Sophie Rees, Christopher Bassford, Jeremy Dale, Zoe Fritz, Frances Griffiths, Helen Parsons, Gavin D. Perkins, Anne Marie Slowther

**Affiliations:** ^1^ Warwick Medical School University of Warwick Coventry UK; ^2^ University Hospitals Coventry and Warwickshire NHS Trust Coventry UK; ^3^ Cambridge University Hospital NHS Trust Cambridge UK; ^4^ University of the Witwatersrand Johannesburg South Africa; ^5^ Heartlands Hospital University Hospitals Birmingham Birmingham UK

**Keywords:** acceptability, decision making, feasibility, intensive care, intervention, implementation

## Abstract

**Rationale, aims, and objectives:**

Decisions about whether to refer or admit a patient to an intensive care unit (ICU) are clinically, organizationally, and ethically challenging. Many explicit and implicit factors influence these decisions, and there is substantial variability in how they are made, leading to concerns about access to appropriate treatment for critically ill patients. There is currently no guidance to support doctors making these decisions. We developed an intervention with the aim of supporting doctors to make more transparent, consistent, patient‐centred, and ethically justified decisions. This paper reports on the implementation of the intervention at three NHS hospitals in England and evaluates its feasibility in terms of usage, acceptability, and perceived impact on decision making.

**Methods:**

A mixed method study including quantitative assessment of usage and qualitative interviews.

**Results:**

There was moderate uptake of the framework (28.2% of referrals to ICU across all sites during the 3‐month study period). Organizational structure and culture affected implementation. Concerns about increased workload in the context of limited resources were obstacles to its use. Doctors who used it reported a positive impact on decision making, with better articulation and communication of reasons for decisions, and greater attention to patient wishes. The intervention made explicit the uncertainty inherent in these decisions, and this was sometimes challenging. The patient and family information leaflets were not used.

**Conclusions:**

While it is feasible to implement an intervention to improve decision making around referral and admission to ICU, embedding the intervention into existing organizational culture and practice would likely increase adoption. The doctor‐facing elements of the intervention were generally acceptable and were perceived as making ICU decision making more transparent and patient‐centred. While there remained difficulties in articulating the clinical reasoning behind some decisions, the intervention offers an important step towards establishing a more clinically and ethically sound approach to ICU admission.

## 1 INTRODUCTION

Treatment on an intensive care unit (ICU) improves the survival rates for patients with life‐threatening illnesses,^1^ and timely admission to an ICU is associated with better patient outcomes.^2^ However, ICU treatments such as ventilatory and cardiovascular support and renal replacement therapy place a considerable burden on patients: patients may be left with significant physical and psychological morbidity which sometimes persist for many years.^3^ In some cases, the resultant burdens of intensive care treatments may outweigh any potential benefit, and patients may receive greatest benefit by having life‐supporting therapy limited to less invasive treatments that can be provided safely on a ward or from palliative care. Decisions whether or not to opt for treatment on ICU are often made under pressure, while a patient is deteriorating, in the context of clinical uncertainty and with little to no knowledge of what the individual patient might want. Furthermore, ICU treatment is costly and resource intensive with demand regularly outstripping capacity, raising additional organizational and ethical concerns about equity of access.

Despite this complexity, there is currently no specific guidance or framework to assist clinicians with this decision‐making process. The limited available guidance tends to focus on process issues^4^ or high‐level principles such as the need to balance burdens and benefits of treatment.^5^ Empirical studies have found that a wide range of clinical and nonclinical factors influence these decisions, with factors such as the patient's functional status (eg, ability to perform certain activities) and quality of life being assessed from the perspective of the doctor rather than the patient.^6^, ^7^, ^8^, ^9^, ^10^, ^11^, ^12^, ^13^, ^14^, ^15^, ^16^, ^17^, ^18^ There is very little published research on the involvement of patients and those close to them in this decision‐making process, despite this being a principle of good clinical practice.^19^

There is, therefore, a need to support referring teams and ICU doctors who make these decisions in order to consistently achieve more transparent, patient‐centred, and ethically justifiable decisions. To address this, we developed a decision‐support intervention (DSI) to improve the decision‐making process regarding ICU referrals and admissions and tested its feasibility in three hospitals in England. In this paper, we present an evaluation of the intervention in terms of how well it was implemented, its acceptability, and its impact on decision making as perceived by staff at the implementation sites.

## 2 METHODS AND MATERIALS

### 2.1 The intervention

The development and implementation of the intervention was part of a larger project^20^ funded by the National Institute of Health Research which included systematic reviews of the literature, an ethnographic study of the decision‐making process in six NHS hospitals, a specific form of questionnaire survey (choice experiment) completed by ICU consultants and ICU outreach nurses across the United Kingdom, and a stakeholder conference.

**Figure 1:**
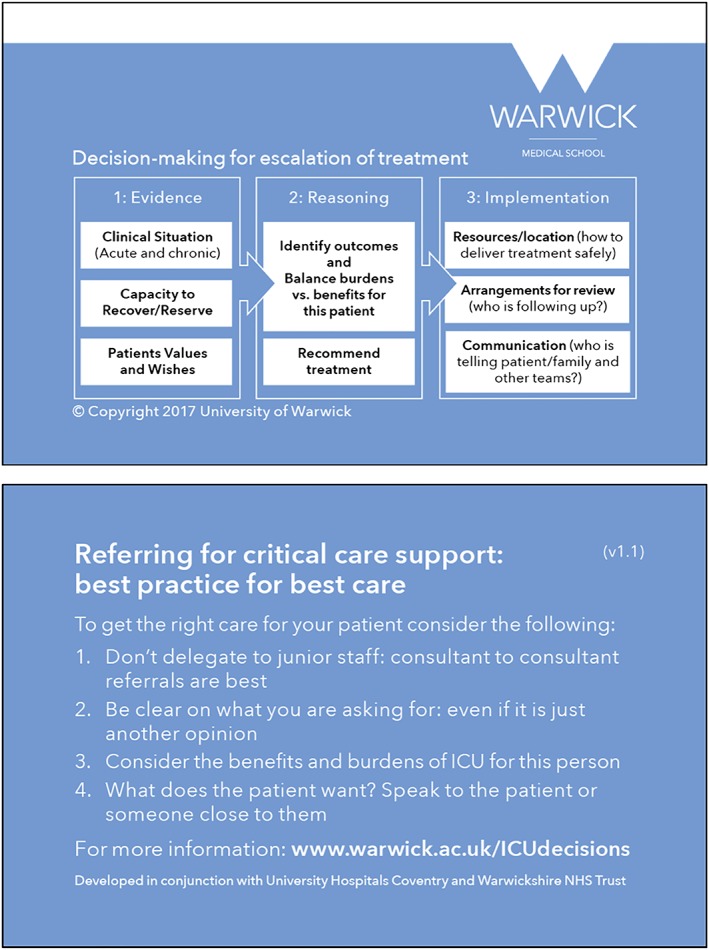
A pocket‐sized summary of the cognitive framework to act as an aide memoire

The decision support intervention drew on data from all elements of the larger project.^21^ It was underpinned by a conceptual “decision support” framework of a patient‐centred, evidence‐based, and ethically justifiable decision process that drew on Accountability for Reasonableness^22^ as its theoretical model. It included the following elements:

1. a referral form to be used by the referring doctor when making a decision whether to *refer* a patient;
2. a decision form to be used by the ICU doctor when making a decision about whether to *admit* a patient;
3. credit card‐sized outlines of the framework as prompts for clinicians (Figure 1);
4. educational materials to be delivered by the hospital's implementation champions; and
5. patient information leaflets (PILs) and family information leaflets (FILs) about ICU to help patients and families better understand what ICU treatment involves.

The referral and decision forms require doctors to document a number of points, including clinical evidence supporting the need for, and ability to benefit from, intensive treatment; evidence of what is known about the patient wishes and values regarding intensive treatment; and the benefits and burdens for this patient of escalating treatment (decision form only). The focus of the decision support framework is on what is likely to be the optimal treatment for this particular patient rather than the binary question of whether or not the patient should be admitted to ICU. Clinicians are asked to document their recommendation for the patient and to explicitly document who has contributed to the decision (ie, referring and ICU team members, patient's family, and/or the patient).

### 2.2 Setting and implementation plan

Having developed the intervention, we drew on the normalization process theory (NPT) literature and ran a workshop with ICU doctors and critical care outreach (CCOR) nurses to inform the implementation strategy.^23^, ^24^

Three NHS hospitals purposively sampled for ICU size (less than 20 ICU beds; 20‐30 ICU beds; more than 30 ICU beds) were recruited as feasibility sites. Each site was asked to identify two members of staff to act as implementation champions, and one chose to have three champions. Champions were ICU consultants (Hospitals A and C), ICU registrars (Hospital B), and CCOR nurses (Hospitals A and C).

The implementation period prior to commencing data collection was set at 8 weeks. Decisions about the specific implementation method were the responsibility of the champions at each site as they were familiar with the structure and processes within their own institution. We held a “train the trainers” event for the champions at each site, providing information about the study, detailed education on the conceptual framework and use of the referral and decision forms. Champions were asked to set up a log for all patients referred to ICU during the study period; one site had an existing log, two introduced it for the study.

### 2.3 Evaluation

A 6‐week data collection period followed the 8 weeks of implementation. A mixed methods approach was used. A single researcher (SR: postdoctoral research fellow with a PhD in health and social studies) collected both quantitative and qualitative data. Quantitative data was collected by examining the records of patients referred to ICU during the data collection period. Qualitative methods included interviews with champions, observation of staff training, and interviews with referring and ICU clinicians during the data collection period. The project was approved by the Coventry and Warwickshire NHS Research Ethics Committee (15/WM/0025), and approval from the National Confidentiality Advisory Group of the Health Research Authority was obtained to access patient records without explicit consent (15/CAG/0116). Research governance approval was provided by each site. In reporting this study, we have applied the Consolidated Criteria for Reporting Qualitative research (COREQ) checklist for in‐depth interviews.^25^

### 2.4 Quantitative data collection

Use of the intervention was recorded by examining the records of all patients identified as having been referred to the three ICUs during the data collection period. For patients who had been referred, we extracted the following data from their clinical records: date, time, and location of referral; doctors making/reviewing the referral; and whether a referral/decision form had been used. If a form was present in the records, the extent of completion was documented.

### 2.5 Qualitative data collection

Interview topic guides were developed for champions and participating doctors. Interviews with implementation champions were conducted regularly throughout the 8‐week implementation period, either face‐to‐face or by telephone. Two instances of the champions delivering the educational materials were observed at each site and field notes taken.

Data from patient records were used to invite referring and ICU doctors to face‐to‐face interviews to explore the intervention's acceptability and impact on their decision making. Participants were sampled according to whether they had used a referral/decision form during the data collection period and to gain a mix of specialties, grades, and admit/not admit decisions (see Table 2). Interviews lasted between 7 and 60 minutes and took place in the hospitals. Written consent was obtained prior to interview. In the interviews, doctors were asked to recall a specific case to avoid hypothetical discussions. The referral and decision support forms were used to prompt their recall. Finally, we carried out “debrief meetings” with the champions at each site to gain further insight into the implementation. All interviews were audio‐recorded, transcribed verbatim, and anonymized.

### 2.6 Data analysis

Data extracted from the patient records were analysed as counts, percentages, and, where there was a continuous variable, means and standard deviation. We looked for associations between patient/doctor/organizational factors and form completion. Chi‐squared tests were used to compare categorical variables, and Student's *t* test was used to compare continuous data. Analyses were carried out by the statistician (HP) using the software R.^26^

All interview transcripts and field notes were entered into NVivo QSR 11^27^ and analysed thematically.^28^, ^29^, ^30^ Initial coding of data was undertaken by SR and checked for consistency by FG (sociologist and GP) using a sample of transcripts. During analysis meetings the team (SR, FG, AS, [ethicist and GP], and CB [ICU consultant]) reviewed codes and emerging themes and checked interpretation against the data, leading to further analysis and refinement of themes until consensus was reached. We used both predefined (from our topic guide) and emergent nodes.

Due to time constraints, the main analysis occurred following data collection, so to check that we had not missed any important perspectives in our interviews, we conducted audio‐recorded debrief meetings with the implementation champions. In these meetings, we explored the emergent themes in our data to aid interpretation and check for missing perspectives. No new perspectives emerged suggesting data saturation had been reached in our interviews.

To integrate the quantitative and qualitative data, we examined both datasets and considered where they converged, complemented, or contradicted one another (triangulation).^31^

## 3 RESULTS

### 3.1 Form usage (compliance with intervention)

Data were extracted from 181 sets of patient records (Table 1). The forms were used in 28.2% of all eligible referrals across the three sites (44.4% at Hospital A, n = 28/63; Hospital B 21.4%, n = 3/14; and Hospital C 19.2%, n = 20/104). The presence of a referral form in the record was associated with a higher likelihood of a decision form being present (16.6% vs 3.3% *P* < .001). We investigated a number of factors with respect to an association with form usage, including patient gender, time of day, and ICU bed availability, but patient age was the only factor that had a statistically significant association with use of the forms: forms were used more often in older patients (*P* < .001, *t* test). See Supporting Information for more detail.

**Table 1:** Patient records

Hospital	Total Referrals Logged	Excluded (Ineligible^a^)	Not Assessed (Unable to Access Notes)	Final Number of Referrals Examined
A	71	8	1	63
B	26	11	1	14
C	236	92	40	104
Total	333	111	42	181

a Eligibility criteria were defined by each site: Hospital A included all unplanned admissions except transplants and between‐hospital transfers; Hospital B opted to include only referrals from haematology/oncology, respiratory, and emergency department (ED) (exc. out‐of‐hospital cardiac arrests); and Hospital C excluded transplant patients, between‐hospital transfers, and referrals directly from theatre.

### 3.2 Implementation

The champions took different approaches to implementation. Hospitals A and C intended to roll the intervention out across the whole site, whereas Hospital B only included referrals from three clinical departments: haematology/oncology, respiratory, and emergency department (ED). Champions at all three sites used the presentations provided by the research team at grand round and departmental meetings, modifying them to be hospital specific. The CCOR champions did not use these resources, preferring to explain the study verbally at team meetings, individually at shift changeovers, and/or utilizing a group message service to remind colleagues about the study. The champions placed boxes containing forms in prominent positions on hospital wards and the ICU.

The champions described several challenges in achieving implementation.

Reaching target groups to raise awareness and to deliver training on the intervention within the time period (8 weeks) was challenging, particularly at the two larger hospitals. The champions at Hospital B decided to limit the use of referral forms to three clinical areas to overcome this. In practice, however, this meant that ICU doctors at this site forgot about or disregarded the forms in between referrals from these three areas.

The status and credibility of champions within a hospital influenced the success of embedding the intervention. While the ICU consultants at two sites felt comfortable in the champion role, the registrar champions at the third expressed some uneasiness over their credibility as change agents:

It's difficult to disagree with people who are our consultants and are signing our feedback form; there's only so much opposition or contrasting opinion I can vocalise. (Champion 2, Hospital B)

Two hospitals (A and C) had CCOR teams (specialist nurses who provide support for patients with, or at risk of, critical illness who are on wards outside the ICU). At these sites, the CCOR team led by the CCOR champion(s), raised the profile of the intervention and could provide the referral form to referrers early in their decision‐making process. Hospital B relied solely on ICU doctors prompting about the form at the time of referral, when the decision to refer had already been made. At Hospital A, the clinical director of one specialty championed the use of the referral form in their unit, integrating it into their admission process by adding a prompt to their electronic admission document, which facilitated its usage.

Motivating the ICU doctors was also a challenge for the champions.

I expected more resistance from [referrers] … it's the ICU doctors I can't understand not filling in the forms. (Champion 3, Hospital C)

At Hospital A, the champions used a “league table” to harness the competitive spirit between the doctors, challenging them to achieve the highest percentage of form use in the referrals and decisions they made.

The champions at Hospital C felt that the intervention fitted well with their already‐existing activities in relation to recognizing and improving advance decision making for deteriorating adults. For example, their hospital had recently embedded daily “safety huddles” on every ward, which included discussion of patients who were not for resuscitation and who were or were at risk of deteriorating. They were hopeful that this would improve the chances of embedding the intervention given the significant crossover.

### 3.3 Acceptability of the intervention

To explore acceptability, 19 referring doctors and 20 ICU doctors were interviewed of the 50 we approached. The level of seniority of participating doctors, and the range of specialties of referring doctors is shown in table two, together with data on whether participants had used either the referral or decision support form as appropriate. At Hospital C, an opportunistic group interview was undertaken with three CCOR nurses (Table 2).

**Table 2:** Specialty and grade of participating doctors and whether they had ever used the referral or decision support form

Characteristic		Hospital			Total
		A	B	C	
Referring doctors					
Grade	Consultant	3	4	3	10
	Registrar	3	2	2	7
	FY1/2	‐	‐	2	2
Referring specialty	Acute medicine	2	‐	1	3
	ED	1	2	1	4
	Surgery		‐	1	2
	Haematology/Oncology	‐	3	1	4
	Respiratory	1	1	1	3
	Hepatology	‐	‐	1	1
	Geriatrics	‐	‐	1	1
	Medicine	1	‐	‐	1
Number of doctors who had used the referral form	Used	5	2	4	11
	Never used	1	4	3	8
ICU doctors (single specialty)					
Grade	Consultant	3	2	4	9
	Registrar	3	4	4	11
Number of doctors who had used the decision support form	Used	6	2	6	14
	Never used	‐	4	2	6

Abbreviations: ED, emergency department; ICU, intensive care unit.

When doctors had used the form, they generally reported it was easy to use.

It wasn't too much of an onerous process and I thought it was very useful. (Referring doctor 3, Hospital A [Consultant, used form])

However, some worried it would introduce more, or cause duplication of, work and consequently affect their ability to provide patient care.

It's a bit time consuming … When you're, you have the referral bleep, you're seeing lots of patients, ward is busy … (ICU doctor 4, Hospital B [ICU Registrar, used form])

The forms were designed to provide a structured account to be held in the record in place of more generic documentation in the notes. However, doctors appeared uncomfortable if they were not also writing in the formal notes because the form itself was not “Hospital approved documentation”. It was also frequently suggested that incorporating the forms into the hospital's electronic system would facilitate their use and reduce duplication of work and records.

Concerns about workload were particularly noticeable in the ED where doctors described having to make decisions quickly based on little information: decision making was fluid and responsive to new information.

We are too busy to complete forms, simple as that. (Referring doctor 4, Hospital A [ED consultant, never used form])

I'm constantly scoping for new information to update and revise my decision. (Referring doctor 5, Hospital B [ED consultant, used form])

However, one ICU doctor thought the decision‐making framework, prompted by the form, could help to create some space and time, however brief, for the doctor to think through a complex decision.

[ED is] like a busy bazaar, so it [completing the form] adds a little bit of resting quiet normality to an otherwise slightly potentially mad referral. (ICU doctor 6, Hospital B [Registrar, used form])

A small number of consultants, both referring and reviewing, commented that the requirement to complete a form, or follow an explicit framework, called into question their clinical judgment and expertise. They felt this to be unnecessary:

I don't need it to help me make a decision because like otherwise what have I been doing for the last ten years? (ICU doctor 1, Hospital B [Consultant, never used form])

### 3.4 Impact on decision making

#### 3.4.1 Improving communication and documentation of decision

Several doctors stated the forms helped them to clearly set out a rationale for their decisions, and to communicate their reasoning to colleagues, leading to a shared understanding of the situation.

It makes us as the referrers focus on exactly why we're referring that patient. (Referring doctor 3, Hospital A [Consultant, used form])

However, while the use of forms facilitated articulation and communication of relevant information and reasoning, it did not guarantee it:

The fact that this patient is paranoid and refusing treatment isn't even mentioned [on the completed referral form] and that's the main problem with this patient. (ICU doctor 8, Hospital C [Consultant, used form])

Failing to provide accurate information on a referral form might provoke more irritation than simply failing to mention it in a telephone call or an entry in the medical record.

### 3.5 Considering patient wishes and values

Many doctors noted that the forms prompted them to specifically consider and document the views of their patient, which they would not have routinely done when considering ICU referral/admission.

I think the most important bit was actually speaking to the patient about their wishes … I wouldn't automatically think about … It prompted me to mention to him or at least check with him that he was happy with the plan to refer him to ITU. (Referring doctor 6, Hospital B [Registrar, used form])

It also makes you discuss things with the patient. Often we refer people when we've not even asked them if that's something that they'd like to undergo! So that's another useful part of the form as well. (Referring doctor 3, Hospital A [Consultant, used form])

I can think of a patient actually downstairs whereby the form prompted them to go and have that discussion. And in fact that patient didn't come to ICU. (ICU doctor 3, Hospital C [ICU Consultant, used form])

However, there was some doubt about how well a patient can communicate their views about an admission to ICU given that these decisions are often made in emergency situations when patients have little understanding of the nature of intensive care treatment, and there is a limited opportunity to explain it or consider alternative options.

There is only a small proportion [of patients] in which these values and wishes are expressed in an informed way. (Referring doctor 2, Hospital B [Consultant, never used form])

The intervention included PILs/FILs that had been codesigned with substantial input from our patient advisory group and a stakeholder conference. These provided clearly presented information about what an ICU is and what kinds of treatments might be provided. However, none of the sites managed to embed these, and interview participants were usually unaware of their existence. They agreed with the principle of providing information to patients and families, but highlighted the difficulty of actually using the leaflets at the time a referral or review took place, when a patient is already deteriorating.

You don't want to be making that decision when you're unwell, you want to make that decision beforehand and have thought about it before the event arises … I would probably prefer to use them in the less acute situation. (Referring doctor 6, Hospital B [Registrar, used form])

Champions said they were unable to implement the PILs/FILs because they were focusing their efforts on embedding use of the clinician forms, which was a challenging enough task. Champions also expressed concern that the leaflets might cause distress to patients and families if provided without appropriate explanation and support.

### 3.6 Making the decision

The decision forms required ICU doctors to document the “benefits and burdens” of ICU treatment for the particular patient, to encourage explicit consideration of advantages and disadvantages of escalating care in coming to a decision. The “benefits” were documented in 86.1% (n = 31/36) of cases, and the “burdens” in 69.4% (n = 25/36). We explored these relatively low completion rates, particularly of the burdens, in the interviews. Some doctors felt the burdens of ICU treatment were the same for all patients and thus did not need to be specified:

You might as well print the burdens of intensive care on the form because y'know largely they're going to be the same. (ICU doctor 3, Hospital C [ICU Consultant, used form])

Others reflected that the very complex and personalized nature of the decision, including weighing benefits and burdens, meant it was difficult to articulate and summarize on a form:

It's got to be a decision taken within the context of that clinical case … but it's quite, it is difficult to put that in writing. (ICU doctor 5, Hospital C [ICU Consultant, used form])

The burdens are quite hard to articulate, although we know they're there and we know they're profound. (ICU doctor 2, Hospital C [ICU Registrar, used form])

The aim of the forms was to aid decision making in real time, requiring completion during the decision‐making process, but in practice, they were often completed after a decision had been made, which meant doctors were more reluctant to document the burdens of a treatment they had already recommended or implemented.

Yes it comes with burdens but if you've decided to take the patient to intensive care it's quite hard to … to document that I think. (ICU doctor 3, Hospital C [ICU Consultant, used form])

## 4 DISCUSSION

This study has demonstrated the feasibility of implementing an intervention to facilitate a transparent and ethically justifiable decision‐making process around ICU admission in three contrasting hospital settings. However, uptake of the intervention across the sites was variable, from just under 20% at one site to 44% at another, and champions struggled to implement the PILs/FILs. Clinicians who used the referral and decision forms perceived them as acceptable and impacting positively on practice. Observation and interview data identified several challenges to and facilitators of the intervention uptake. A key message was the need for organizational endorsement and adoption of the forms as official hospital documentation if the intervention was to become embedded in day‐to‐day clinical practice. Implementation champions recommended an electronic format for the forms to facilitate and prompt its integration into decision‐making and hospital documentation systems. Despite the challenges encountered, two of the three sites expressed an interest in taking the intervention or a variation of it forward as a service development.

### 4.1 Implementation

May and colleagues have argued that when implementing an intervention, contextual factors should be seen as the “normal conditions” of practice into which the intervention needs to be integrated, rather than as confounders to be eliminated.^32^ All our sites shared the context of a busy NHS hospital, which created challenges for the implementation champions both in finding time to promote the intervention and engaging their referring and ICU colleagues in both learning about and implementing the intervention. However, the size of the organization made a difference. For example, the highest uptake was at our smallest site, which was probably related to the ability of the champions to reach key individuals more easily and to maintain continuing awareness of the intervention among a smaller pool of colleagues. The presence of a CCOR team also facilitated intervention uptake. The CCOR are involved in the early identification of patients who may subsequently be referred to ICU and often act as a link between referring teams and ICU teams. The intervention therefore fitted directly into much of their daily practice, so they were well placed to remind both referring and ICU doctors to use the forms. Timely reminders to referring doctors also encouraged appropriate use of the referral forms rather than a post hoc request to document the reasons for a referral that had already been made.

May also suggests that the plasticity of an intervention contributes to the ease with which it can be successfully integrated into different contexts.^24^, ^33^ The educational component of the intervention had some plasticity in that its format and the presentation of intervention materials could be modified to fit with established educational opportunities within each hospital. There was also some plasticity in the mode of delivery of the forms to referring teams. Being able to upload the referral form and include it in one specialty team's electronic referral system substantially improved knowledge of and uptake of the referral form. However, the content of the referral and decision support forms could not be amended, and this rigidity meant champions could not respond to criticisms or suggestions for change from their colleagues during implementation. Future implementation work will need to consider the balance between need for flexibility and maintenance of the core components of the framework.

A key concern of health care professionals regarding new interventions at organizational level relates to increased workload and duplication.^24^ These concerns were expressed by doctors in our study, but interview data from our champions suggested possible ways to address this. Incorporating the forms into the hospital electronic record system would identify them as permanent formats for recording patient information removing the need for backup paper notes. This was supported by the evidence from the single specialty team noted above. We had developed a prototype electronic version of the intervention for use during the implementation study, but none of the sites were able to make use of this within the time frame of the project. This kind of integration needs a much longer lead in time and a commitment by the organization to commit to the intervention on a medium‐ to long‐term basis, a challenge when its effectiveness and acceptability is unknown. The importance of senior management support in generating a positive implementation climate, the intervention's fit with organizational and professional values, and the integration of the intervention with other innovations within the organization are all recognized as facilitators of effective implementation.^34^, ^35^, ^36^ While there was some evidence of senior management support for the intervention, the intervention was not seen as integrated with a wider programme of innovation. More attention to these aspects of implementation will be required to embed a future version of the intervention into routine NHS practice.

The doctors in our study were required to change their practice, both in terms of documenting their decisions and in structuring their decision‐making process. Some interpreted the intervention as an implicit criticism of their expertise and resisted any suggestion that current practice needed changing. This normative restructuring is a challenge for implementation of new interventions and champions need to have sufficient authority or respect both within the organization and within the relevant teams to legitimize its use.^24^, ^33^ Implementation champions who were ICU consultants or senior outreach nurses found this easier than our registrar champions because of their position in the organizational hierarchy and their existing relationship with colleagues, but absence of champions within referring teams made integration of referral forms more difficult.

### 4.2 Impact of the intervention on decision making

As there are currently no validated tools for evaluating ethical decision making in real‐time clinical practice, we were unable to formally evaluate the referral and admission decisions. However, the interviews with ICU and referring doctors revealed that the intervention had influenced practice in a number of ways including, improving clarity of documentation and communication of decisions with colleagues, considering patient's values and wishes, and thinking through the decision process in a structured way. A key aim of the intervention was to support clinicians in making ethically justifiable decisions based on considering and balancing all relevant information.

The literature on diagnostic error provides insight into how clinicians make decisions in relation to patient treatment. It suggests decision makers tend to rely on heuristics, tacit knowledge, and contextual experience when making complex decisions.^37^, ^38^, ^39^, ^40^, ^41^ In the context of intensive care, studies have shown clinicians' predictions of poor outcome for certain patient groups to be unreliable when they rely on their clinical assessment over objective measurements, suggesting an element of cognitive bias.^42^, ^43^ Effective educational and work place interventions to counteract cognitive bias and improve critical thinking are underresearched but include prompts for reorganization of knowledge and reflection on the evidence.^44^, ^45^ The intervention encouraged a structured approach to information gathering and reflection on decision making through prompts to explicitly weigh burdens and benefits of ICU treatment for the particular patient. However, it was clear from the analysis of completed forms and the interview data that doctors had difficulty articulating and documenting the reasoning process underlying their decisions. Better training in use of the form may have improved their engagement with this element of the process, but it is also possible that familiarity and habituation with using the form over time is a necessary step in reframing the cognitive process to include more explicit reflection on burdens and benefits. Changing a person's often long‐established cognitive mechanisms for decision making is a challenge, especially in just a few weeks. Decisions regarding admission to intensive care often involve a high level of uncertainty; information is lacking because of the urgency of the situation, prognosis is difficult to estimate, and the patient cannot communicate their wishes. For our participants, this uncertainty was reflected in their reluctance to commit to documentation of specific burdens and benefits of ICU treatment for the patient. As Cummings et al note, “interventions aimed at improving care for patients facing clinical uncertainty can be difficult to integrate due to the very nature of complexity that exists for these patients and their clinicians.”^24^

### 4.3 Strengths and weaknesses

The use of quantitative and qualitative methods meant we were able to triangulate the data.^46^ We achieved diversity in our sample in terms of clinician specialty and grade, and sites reflected a range of NHS ICU settings. Specific cases were used during the interviews to avoid hypothetical scenarios or generalizations as much as possible. The position of the interviewer as a nonclinician meant that assumptions about patient care and decision making were probed for clarity and understanding without preconceptions about what “normal practice” might be. Doctors might have found it easier to discuss cases with a fellow clinician, but conversely, they may have felt less comfortable discussing or admitting to uncertainty with a clinical colleague.

Eight weeks may not have been a long enough period to embed the intervention in clinical practice, a point that was raised by our champions. However, a longer period may not have addressed the intervention being perceived as a time‐limited research project, rather than an organization‐driven quality improvement. We found the retrieval of clinical records within the study time period challenging. Many doctors did not write their names clearly in the patient record, so we were unable to identify them to request an interview. Poor documentation in patient records also resulted in missing data, so our quantitative results should be interpreted with caution. We were nevertheless able to identify differences in form use, which were supported by our qualitative findings. We did not observe informal education/training, nor did we observe referrals as they happened. If the intervention is implemented more widely, future research should explore the process from the perspective of patients and families, whose voices are currently missing from the data.

## 5 CONCLUSION

We have demonstrated that it is feasible to implement an intervention to support decision making around referral and admission to intensive care. When used, the decision support framework appeared to improve practice in terms of encouraging more transparent (better documented and communicated) and patient‐centred decision making (greater attention to patient wishes and values), and improved communication between staff, but clinicians still found it difficult to articulate the process of balancing burdens and benefits of treatment. However, to achieve full implementation in practice, the decision support framework for referral and admission decisions needs to be embedded within normal referral pathways so that they become a routine aspect of practice for decision makers. We suggest that future implementation of the intervention should take place over a longer period with concurrent evaluation, ideally as a part of an action research^47^, ^48^ or a quality improvement^49^ approach. This would require high‐level organization support and allow an iterative process within each setting, allowing for response and adaptation to obstacles to embedding.

## FUNDING INFORMATION

This article presents independent research funded by the National Institute for Health Research (NIHR) under the Health Services and Delivery Research programme (Ref. 13/10/14). The views expressed in this publication are those of the authors and not necessarily those of the NIHR or the Department of Health and Social Care.

## CONFLICT OF INTEREST

A.S., F.G., J.D., and C.B. received grants from the UK National Institute of Health Research during the conduct of the study.

## ETHICAL APPROVAL

The study was approved by the National Research Ethics Service Committee West Midlands—Black Country (Ref. 15/WM/0025). Written consent to participate, including consent for use of anonymized quotations in publications, was obtained from all interview participants.

Approval from the Confidential Advisory Group of the Health Research Authority was obtained to review patient records without consent for analysis of form completion.

## Supporting information

Data S1 Supporting information
