# Supplementary material for: Implementing an intervention to improve decision making around referral and admission to intensive care: Results of feasibility testing in three NHS hospitals
Source: J Eval Clin Pract. 2019 May 17;26(1):56–65. doi: 10.1111/jep.13167 (PMC7003751; doi:10.1111/jep.13167)
Supplement: Supplementary file 1 — Data S1 Supporting information [file JEP-26-56-s001.docx]

## Referral form

INSERT LOGO **TO BE COMPLETED BY REFERRING TEAM**


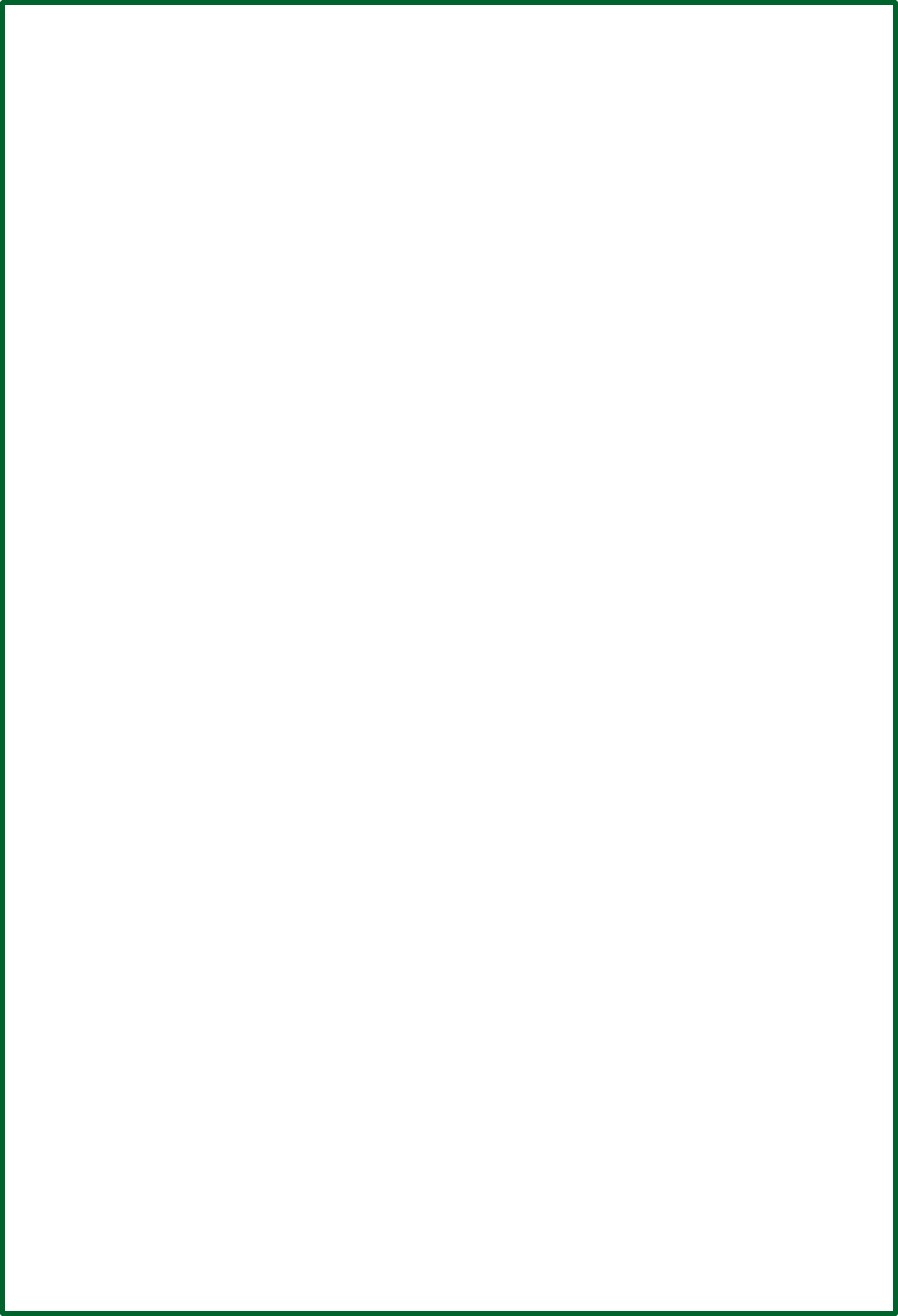


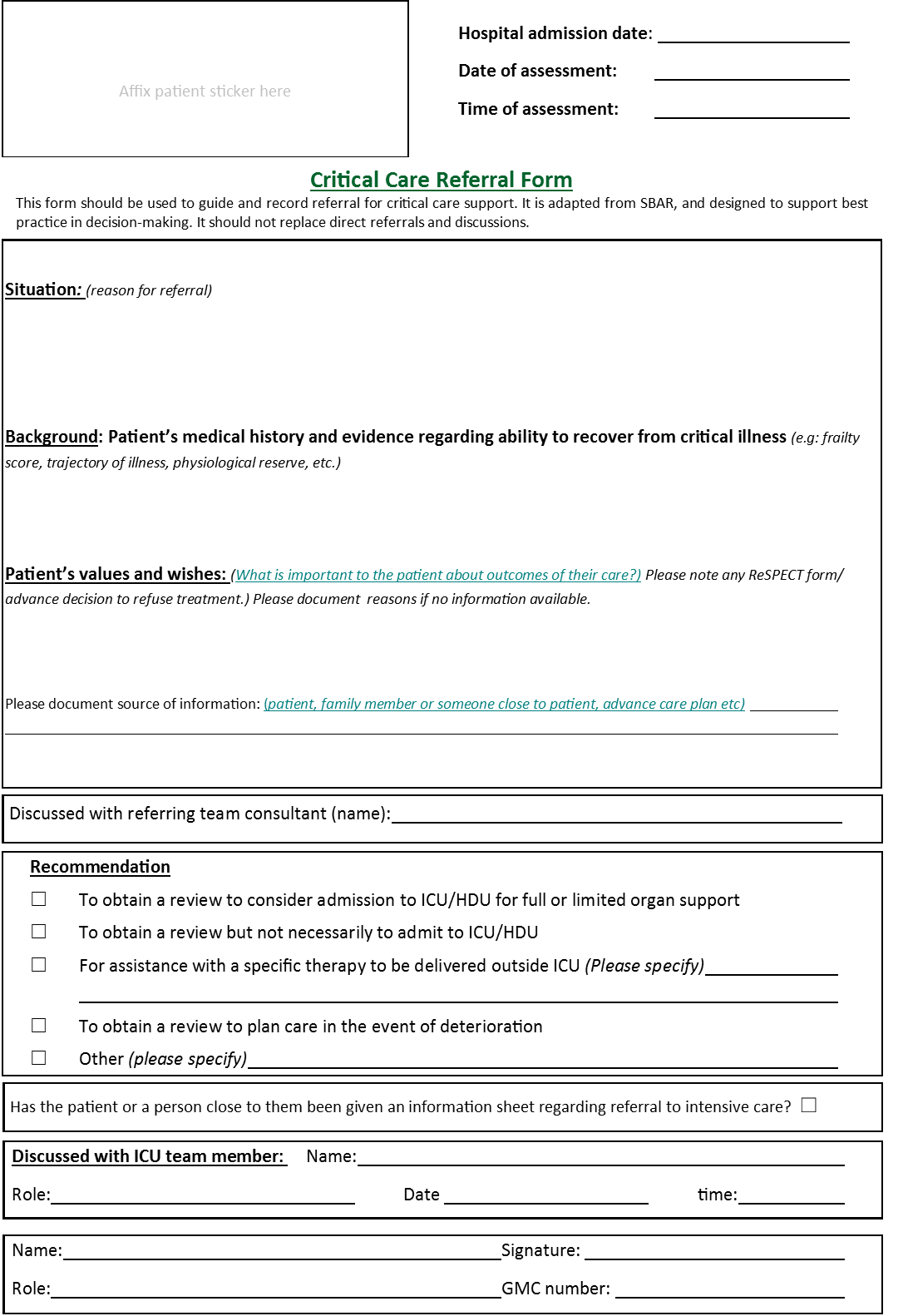


Decision-making for ICU admissions This project was funded by the NIHR HS&DR programme (project number 13/10/14)

Referral form v1.3 25.04.2017 Developed in conjunction with University Hospitals Coventry and Warwickshire NHS Trust © Copyright 2017 University of Warwick

## Decision form

INSERT LOGO  **TO BE COMPLETED BY ICU TEAM**

**Evidence*: Clinical*** *(factors in patient’s acute condition and long term health relevant to decision about escalating treatment)*

**Evidence: Ability to recover from this critical illness based on evidence**  *(e.g****:*** *functional reserve, trajectory of illness, exercise capacity, dependence, self-reported QoL, frailty score)*

**Evidence: Patient values and wishes**  *(what is important to the patient with regard to their treatment and the potential out-comes? Please note ReSPECT form/advance decision to refuse treatment if available.) If no information is available please say why.*

*Please document source of this information: (patient, family or someone close to patient, advance care plan etc)*

*_____________*

**Critical Care: Decision-support Form**

This form can be used to guide and record the decision-making process regarding the critical care support a critically ill pa-tient should receive. It is designed to support best practice in decision-making.

Affix patient sticker here

**Hospital admission date**:

**Date of assessment:**
**Time of assessment:**

**Assessment number** *(for repeat assessments)*

Decision-making for ICU admissions This project was funded by the NIHR HS&DR programme (project number 13/10/14)

Decision form v1.2 10.03.2017 Developed in conjunction with University Hospitals Coventry and Warwickshire NHS Trust © Copyright 2017 University of Warwick


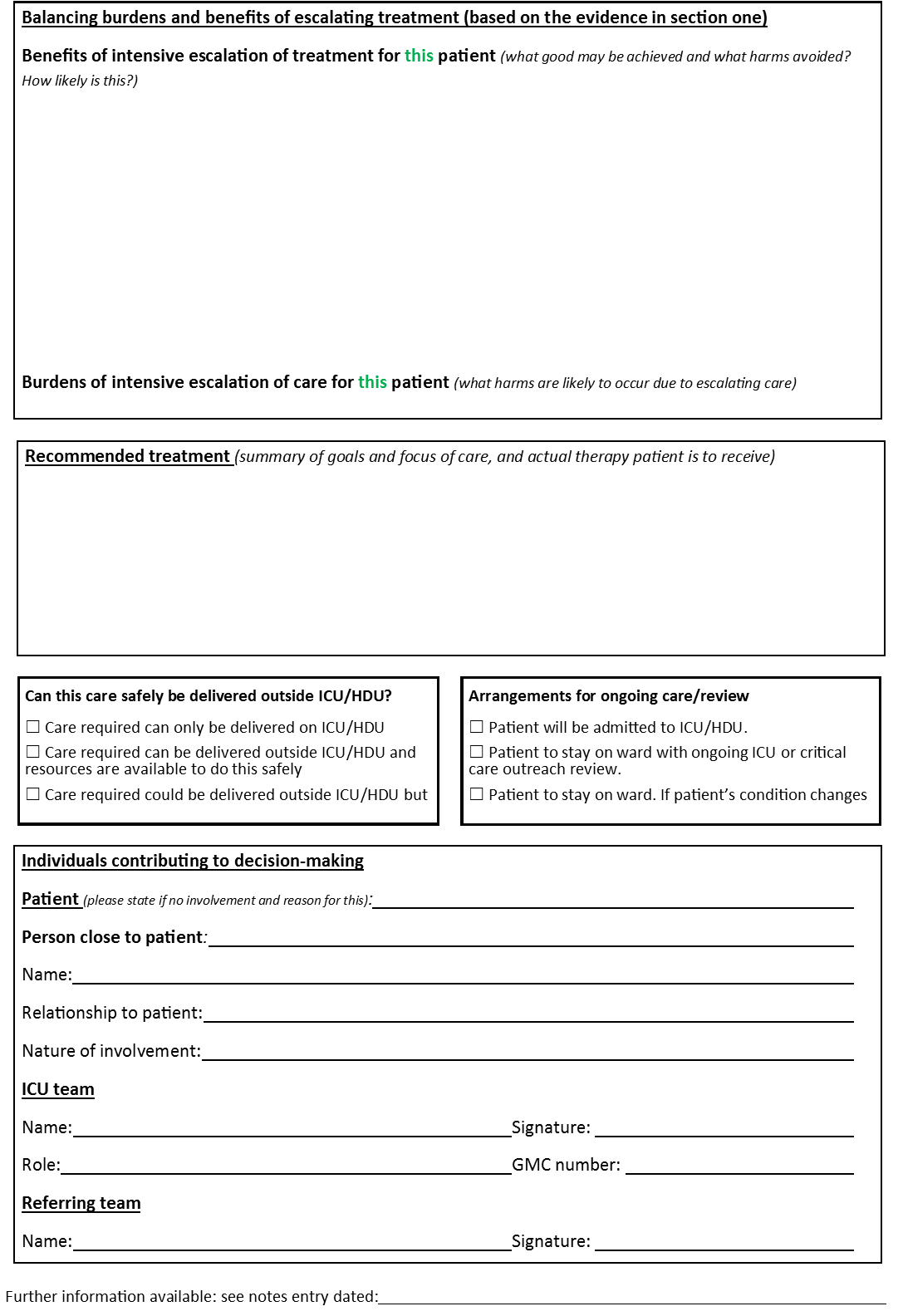


Decision-making for ICU admissions This project was funded by the NIHR HS&DR programme (project number 13/10/14)

Decision form v1.2 10.03.2017 Developed in conjunction with University Hospitals Coventry and Warwickshire NHS Trust © Copyright 2017 University of Warwick

## Implementation Champion Interview Topic guide:

| 1. Question/topic | 1. Prompts/follow-up questions |
| --- | --- |
| 1. How are you getting on with [name of DSF]? 2. What has happened since you started introducing [DSF]/ we last spoke? |  |
| 1. Have you come up against any problems in the implementation? | 1. How have you resolved these problems? 2. Last time you mentioned ____________, how have you dealt with this? |
| 1. Is there anything which you have found has made it easier to implement? | 1. Is there anything that has gone really smoothly? In what way did that make it easier? |
| 1. Have you needed to make any changes to the DSF or its delivery? |  |
| 1. Is there anything else you would like to mention? |  |
| 1. PIL/FILs |  |

## Clinician Interview Topic Guide

| Can you explain your role and position within the hospital? |  |
| --- | --- |
| Have you used [DSF]? | How often? |
| Tell me about the last case when you considered admission/received a referral.  …. And the one before that | How did you use the DSF? Where did it fit in the process?  How did you find using the DSF?  Did it change the way you approached or thought about decisions about ICU referral/admission (use as appropriate)? |
| Why did you use it? | Consultants – role models – using it to teach others?  Registrars/trainees – doing the weighing up or only documenting? |
| Is there anything you did not like about the DSF? | Why?  What would you change?  If you had questions about completing the DSF, did you know who to ask? Did you ask for help? |
| Is there anything specific you liked about the DSF? | Why? How could it be improved? |
| Would you like to continue using it if it were available? | Why/not?  How could we (have) encourage(d) use of it? |
| Did you give out a PIL/FIL? |  |
